# pERK-dependent defective TCR-mediated activation of CD4^+^ T cells in end-stage renal disease patients

**DOI:** 10.1186/s12979-017-0096-1

**Published:** 2017-06-19

**Authors:** Ling Huang, Nicolle H. R. Litjens, Nynke M. Kannegieter, Mariska Klepper, Carla C. Baan, Michiel G. H. Betjes

**Affiliations:** Department of Internal Medicine, Section Nephrology and Transplantation, Erasmus University Medical Center, Rotterdam, the Netherlands

**Keywords:** ESRD, T cells, ERK, p38, DUSP 6, MAPK

## Abstract

**Background:**

Patients with end-stage renal disease (ESRD) have an impaired immune response with a prematurely aged T-cell system. Mitogen-activated protein kinases (MAPKs) including extracellular signal-regulated kinase (ERK) and p38, regulate diverse cellular programs by transferring extracellular signals into an intracellular response. T cell receptor (TCR)-induced phosphorylation of ERK (pERK) may show an age-associated decline, which can be reversed by inhibiting dual specific phosphatase (DUSP) 6, a cytoplasmic phosphatase with substrate specificity to dephosphorylate pERK. The aim of this study was to assess whether ESRD affects TCR-mediated signaling and explore possibilities for intervening in ESRD-associated defective T-cell mediated immunity.

**Results:**

An age-associated decline in TCR-induced pERK-levels was observed in the different CD4^+^ (*P* < 0.05), but not CD8^+^, T-cell subsets from healthy individuals (HI). Interestingly, pERK-levels of CD4^+^ T-cell subsets from young ESRD patients were in between young and elderly HI. A differentiation-associated decline in TCR-induced ERK and p38 phosphorylation was observed in T cells, although TCR-induced p38 phosphorylation was not significantly affected by age and/or ESRD. Frequencies of TCR-induced CD69-expressing CD4^+^ T cells declined with age and were positively associated with pERK. In addition, an age-associated tendency of increased expression of DUSP6 was observed in CD4^+^ T cells of HI and DUSP6 expression in young ESRD patients was similar to old HI. Inhibition of DUSP6 significantly increased TCR-induced pERK-levels of CD4^+^ T cells in young and elderly ESRD patients, and elderly HI.

**Conclusions:**

TCR-mediated phosphorylation of ERK is affected in young ESRD patients consistent with the concept of premature immunological T cell ageing. Inhibition of DUSP6 specific for pERK might be a potential intervention enhancing T-cell mediated immunity in ESRD patients.

**Electronic supplementary material:**

The online version of this article (doi:10.1186/s12979-017-0096-1) contains supplementary material, which is available to authorized users.

## Background

ESRD patients have a defective T-cell mediated immune system that is clinically characterized by an increased risk of a variety of infections [1, 2] and impaired response of vaccination [3–7]. Infections are the second leading cause of mortality following cardiovascular disease and a major cause of morbidity in ESRD patients [8].

Uremia-associated T-cell defects closely resemble premature immunological T-cell ageing [9]. ESRD patients have a discrepancy of 15–20 years between the immunological T-cell age and their chronological age [10]. Declined thymic output, more differentiated memory T cells, T cells lacking co-stimulatory molecules like CD28, skewed T cell receptor (TCR) Vβ repertoire diversity and shorter telomere length are observed in ESRD patients compared to age-matched healthy individuals (HI) [11].

TCR-induced signaling mediates clonal (positive or negative) selection of thymocytes in the thymus and initiates T cell immune responses in the periphery, consisting of T cell proliferation and differentiation [12]. The mitogen-activated protein kinase (MAPK) pathway is one of the major pathways induced upon TCR stimulation [13]. Activation of MAPK is mediated by phosphorylation of MAPK and downregulated by MAPK phosphatase resulting in inactive MAPK [14]. In particular, the extracellular signal-regulated kinase (ERK) pathway is one of the important MAPK pathways. Phosphorylation of ERK can reduce sensitivity of cells to apoptosis and promote cell proliferation [15]. ERK activity controls the positive feedback loop in the TCR-induced activation cascade and reduced ERK activity affects signal strength and activation of T cells [16, 17]. Reduced phosphorylation of ERK is associated with decreased interleukin-2 (IL-2) production [18] and vice versa [19]. Dual specific phosphatases (DUSPs) represents a family of phosphatases that dephosphorylate phosphor-threonine and phosphor-tyrosine residues on MAPK and that are pivotal regulators of MAPK activities. DUSP6 is a cytoplasmic phosphatase with substrate specificity to dephosphorylate pERK [20]. Ageing is also associated with defective signaling pathways [21, 22]. Recently it was shown that decreased phosphorylation of ERK in naive CD4^+^ T cells from elderly HI was associated with more time to build up the required signaling strength following stimulation compared to those from young HI. This decreased phosphorylation of ERK can be overcome by inhibiting DUSP6 [16].

P38 is another pivotal protein in the MAPK pathway [23] and of interest with respect to age-related changes is T cell activation. Most stimuli, including engagement of TCR, costimulatory receptors, inflammation, stress, growth factors, as well as DNA damage induce phosphorylation of p38 by various pathways [24, 25]. Although phosphorylation of ERK and p38 from T cells share some upstream molecules after triggering of TCR, such as phosphorylation of CD3 zeta-chain associated protein kinase of 70 kDa (ZAP70) [26], they each have their unique upstream MAPK kinases (MKKs) [14]. Highly differentiated CD4^+^ T cells lacking expression of CD28 are accumulated in elderly healthy individuals [27], patients with ESRD [28], following chronic viral infection [29], and also in patients suffering from autoimmune disease [30]. Senescent CD27^−^CD28^−^CD4^+^ T cells employ an MKK-independent mechanism for phosphorylating p38 and depend on 5′ adenosine monophosphate-activated protein kinase (AMPK) and transforming growth factor-β-activated protein kinase 1(TAK1)-binding protein 1(TAB1) ex vivo [31].

Little is known as to how MAPK signaling pathways in ESRD patients. Understanding MAPK signaling in ESRD patients may increase knowledge about mechanisms of uremia-associated impaired T-cell mediated immunity and offer possibilities for intervention. Here, we demonstrate that TCR-induced phosphorylation of ERK, and not p38, in CD4^+^ T cells decreases with age and T cell differentiation. This pathway is specifically affected in young ESRD patients and at the level of elderly healthy individuals, compatible with the concept of premature immunological T cell ageing in patients with renal failure. In addition, inhibition of DUSP6 may offer a potential intervention for improving T-cell mediated immunity in ESRD patients.

## Methods

### Study population

In line with our previous studies, young and elderly patients groups were defined based on their chronological age [32, 33]. Twenty-four stable ESRD patients, defined as having a glomerular filtration rate of ≤15 ml/min with or without renal replacement therapy (RRT; i.e. dialysis) and 24 HI were included (Study population characteristics are described in Table 1) at the outpatient clinic. Patients with any clinical or laboratory evidence of acute bacterial or viral infection, malignancy, immunosuppressive drug treatment within 28 days prior to transplantation (except for glucocorticoids) were excluded. Lithium-heparinized blood was drawn of ESRD patients and healthy kidney donors. All individuals included gave informed consent and the local medical ethical committee approved the study (METC number: 2012–022). It was conducted according to the principles of Declaration of Helsinki and in compliance with International Conference on Harmonization/Good Clinical Practice regulations.

**Table 1:** Clinical characteristics of the study population

	HI	ESRD patients	*P* value
Number of individuals	24	24	
Age groups (years; mean ± SD)			
young	29,4 ± 5,6	34,6 ± 8,0	ns
elderly	70,5 ± 5,8	70,8 ± 4,2	ns
Sex (% male)	50	79,2	ns
CMV IgG serostatus (% pos)	62,5	62,5	ns
RRT (number; %)		11; (45,8%)	
Duration of RRT in months (median with range)		22 (1—37)	
Hemodialysis (number)		7	
Peritoneal dialysis (number)		4	
Underlying kidney disease (number; %)			
atherosclerosis/hypertensive nephropathy		8; (33%)	
primary glomerulopathy		4; (17%)	
Diabetic nephropathy		6; (25%)	
congenital disorder		3; (13%)	
others		2; (8%)	
unknown		1; (4%)	

### PBMCs preparation

Peripheral blood mononuclear cells (PBMCs) were isolated from peripheral blood from HI and ESRD patients as described previously [34] and then cryopreserved for further analysis.

### Phosphorylation-specific flow cytometry

PBMCs were stained with eFluor-450-labeled anti-CD7 (eBioscience, Vienna, Austria), allophycocyanin-Cy7 (APC-Cy7)-labeled anti-CD8 (BD, Erembodegem, Belgium), Brilliant Violet (BV)-510-labeled anti-CD16 (BD) and fluorescein isothiocyanate (FITC)-labeled anti-CCR7 (R&D system, Uithoorn, the Netherlands) for 30 min at room temperature. Then 1 million PBMCs/50 μl were prepared for stimulation by labeling cells with 20 μg/ml mouse anti-human CD3 (BD) and mouse anti-human CD28 (BD) each on ice for 20 min, followed by an incubation with goat-anti mouse IgG (BD) for cross-linking on ice for 20 min. Stimulation was initiated by transferring cells to a 37 °C water bath for 10 min. Cells were fixed using Cytofix (BD) at 37 °C for 10 min and then permeabilized in 70% methanol at -20 °C for 30 min. Subsequently, cells were stained with peridinin chlorophyll (PerCP)-labeled anti-CD4 (BD), phycoerythrin (PE)-Cy7-labeled anti-CD45RO (BioLegend, Uithoorn, Netherlands), PE-labeled anti-phospho-p38MAPK (pT180/pY182) (BD), and Alexa Fluor (AF) 647-labeled anti-phospho-ERK1/2 (pThr202/pTyr204) (BD). Phosphorylation was measured on a BD FACSCanto II flow cytometer (BD) and data were analyzed by Kaluza™ software (Beckman Coulter, Woerden, Netherlands). Median fluorescence intensity (MFI) of phosphorylated ERK or p38, generated by Kaluza™, were multiplied by 256 to make them comparable to the data analyzed by FACS Diva software (linear value instead of log-transformed value) (BD). The MFI obtained for anti-CD3/anti-CD28-stimulation were corrected by subtracting the MFI of the unstimulated condition.

### CD69 and IL-2 measurement

PBMCs were either not or stimulated with anti-CD3/anti-CD28 T-cell expander beads (Invitrogen Dynal, Oslo, Norway) at different ratios, i.e. 1 cell / 0.1 bead, 1 cell / 0.5 bead, 1 cell / 1 bead for 6 h in human culture medium (HCM; RPMI-1640 with GlutaMAX, 10% heat-inactivated pooled human serum and 1% penicillin and streptomycin) (Lonza, Breda, Netherlands) with Golgistop (BD). Then cells were stained with AmCyan-labeled anti-CD3 (BD), Pacific Blue-labeled anti-CD4 (BD), APC-Cyanin 7 (APC-Cy7)-labeled anti-CD8 (BD), APC-labeled anti-CD45RO (BD) and PE-Cy7-labeled anti-CCR7 (R&D Systems) antibodies and a live–dead marker ViaProbe (7-aminoactinomycin D; 7AAD; BD). Upon fixation with FACS lysing solution (BD) and permeabilization using FACS permeabilizing solution 2 (BD), cells were stained intracellular using PE-labeled anti-CD69 (BD) and FITC-labeled anti-IL-2 (BD). Percentages CD69-expressing and IL-2 producing CD4^+^ T cell subsets were evaluated upon measuring the samples on a BD FACSCanto II flow cytometer (BD). Data were analyzed by Kaluza™ software (Beckman Coulter).

### DUSP6/1 inhibition

PBMCs were pre-incubated in HCM including 50 μM (E)-2-benzylidene-3-(cyclohexylamino)-2, 3-dihydro-1 H-inden-1-one (BCI) (Merck – Millipore, Amsterdam, Netherlands) at 37 °C for 1 h. BCI has been shown to be an inhibitor of DUSP6 and DUSP1 activity. PBMCs were subsequently washed 3 times and then stimulated by CD3/CD28 antibodies (as described previously) for 10 mins and then MFI of pERK of BCI-pretreated T cells was measured by Phosphorylation-specific flow cytometry as described previously.

### DUSP6/1 measurement

PBMCs were stained with AmCyan-labeled anti-CD3 (BD), Pacific Blue-labeled anti-CD4 (BD), APC-Cy7-labeled anti-CD8 (Biolegend); APC-labeled anti-CD45RO (BD) and PE-Cy7-labeled anti-CCR7 (R&D Systems) antibodies and 7-AAD for 30 min at 4 °C. Upon fixation and permeabilization using Fix/Perm buffer (eBioscience), 1% bovine serum albumin (Zwijndrecht, Netherlands) was used to block Fc receptors. Then cells were further stained with AF647-labeled anti-DUSP6 (Santa Cruz Biotechnology, Heidelberg, Germany) and PE-labeled anti-DUSP1 (Santa Cruz Biotechnology) for 30 min at 4 °C. MFI of DUSP6 and DUSP1 was measured on a BD FACSCanto II flow cytometer (BD) and data were analyzed using FACS Diva software version 6.1.2 (BD).

### Statistical analyses

Data were analyzed by Graphpad Prism 6 (GraphPad Software, CA, USA). Comparison between two groups (non-parametric data) were using Mann Whitney test. Comparison in multiple groups were using Friedman test followed by Dunn’s Multiple Comparison T test or repeated ANOVA test followed by Bonferroni’s multiple comparison test. Comparison between DUSP6-treated and non-treated conditions was done by Paired T- test. All reported *P*-values are two-sided and were considered statistically significant when *P* < 0.05.

## Results

### Study population

The demographic and clinical characteristics of the study population are given in Table 1. Twelve patients were within the young group (age 22–44 years) and 12 patients belonged to the elderly group (age 66–78 years). Age- and cytomegalovirus (CMV)-matched HI, i.e. 13 young (age 21–40 years) and 11 elderly (age 65–74 years) HI were included for comparison. Approximately half of the ESRD patients received RRT (dialysis) with a median dialysis time of 22 months.

### Decreased ERK phosphorylation in young ESRD patients

**Fig. 1:**
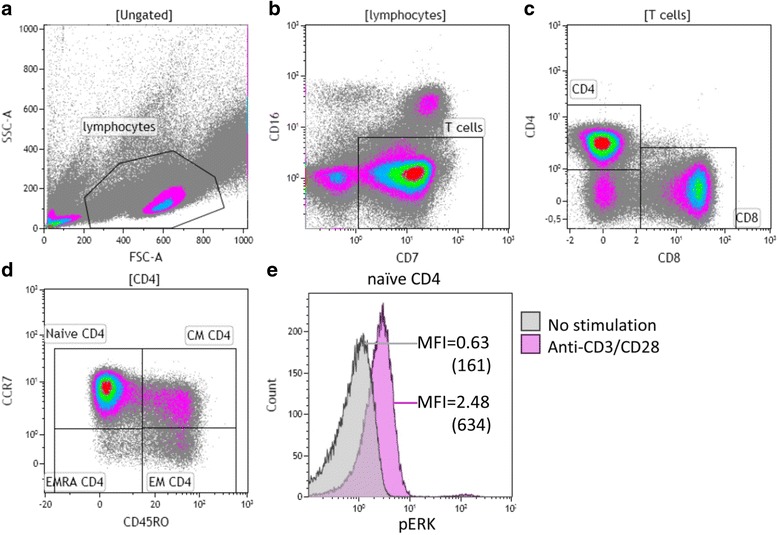
Typical example of the gating strategy for analysis of phosphorylation of ERK (pERK) in naive CD4^+^ T cell subsets. Briefly, (**a**) lymphocytes were identified based on the forward/sideward characteristics followed by (**b**) the selection of CD7^+^ CD16^−^ T cells. **c** These T cells were then dissected into CD4^+^ and CD8^+^ T cells. **d** CCR7 and CD45RO were used to identify naive and different memory subsets within CD4^+^ T cells. Furthermore, (**e**) pERK was measured in naive CD4^+^ T cells without and with CD3/CD28 stimulation, and median fluorescence intensities (MFI) were shown (values multiplied by 256 in *brackets*). A similar gating strategy was employed for phosphorylation of ERK and p38 in all T cells subsets

**Fig. 2:**
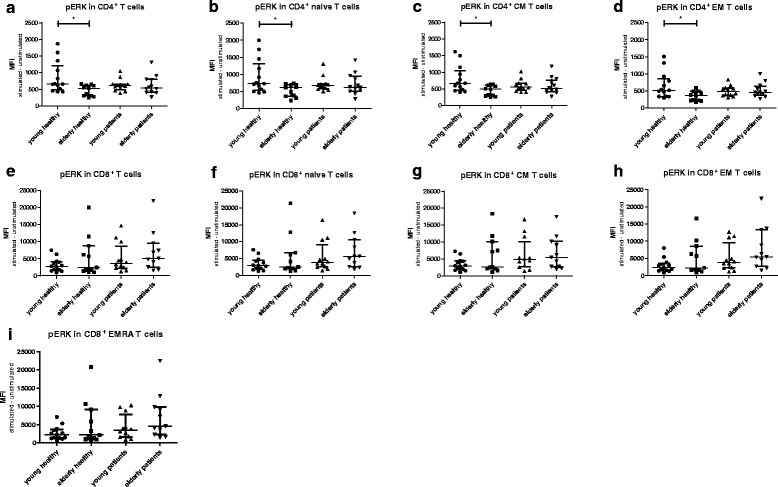
Phosphorylation of ERK in T cells subsets from healthy individuals (HI) and end stage renal disease (ESRD) patients. Phosphorylation of ERK in (**a**) CD4^+^, (**b**) CD4^+^ naive, (**c**) CD4^+^ central memory (CM), (**d**) CD4^+^ effector memory (EM), as well as (**e**) CD8^+^, (**f**) CD8^+^ naive, (**g**) CD8^+^ CM, (**h**) CD8^+^EM, (**i**) CD8^+^ highly differentiated effector T cells (EMRA). *Dots* and *squares* represent young (*n* = 13) and elderly HI (*n* = 11), and *upward*- and *downward-facing triangles* correspond to young (*n* = 12) and elderly (*n* = 12) patients, respectively. *P* value: *< 0.05; Data are given as median with interquartile range

A typical flow cytometric example for analysis of ERK phosphorylation is given in Fig. 1. First, we compared phosphorylation of ERK and p38 between young and elderly HI or young and elderly ESRD patients. A significant age-related lower TCR-mediated phosphorylation of ERK was observed within all CD4^+^ T cell subsets from HI (Fig. 2a–d). For example, the median of MFI value of CD4^+^ phosphorylated ERK (pERK) was 658 in young HI, which was significantly higher than 535 in elderly HI (*p* = 0.015) (Fig. 2a). This trend consistently existed between young and elderly HI when we compared MFI value of pERK in CD4^+^ naive (median 722 vs. 612, *p* = 0.022) (Fig. 2b), CM (median 666 vs. 489, *p* = 0.021) (Fig. 2c) and EM subsets (517 vs. 364, *p* = 0.018) (Fig. 2d). Due to the almost absent EMRA subset within the CD4^+^ T cells, phosphorylation of ERK and p38 within this subset was not evaluated.

**Fig. 3:**
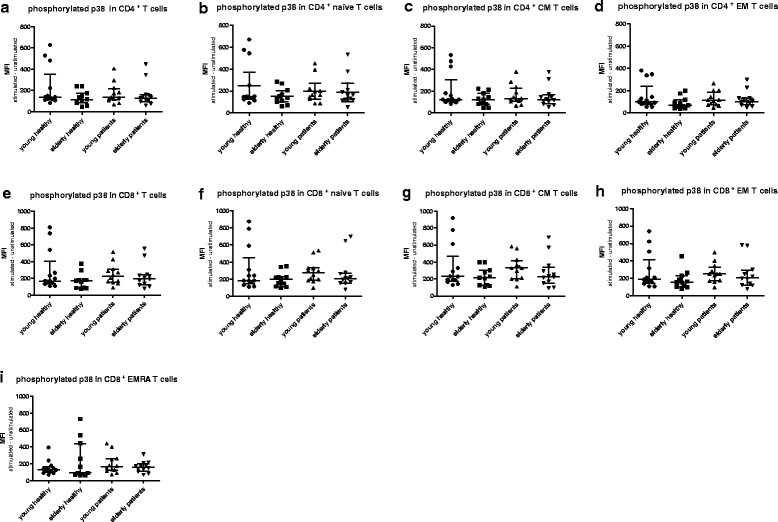
Phosphorylation of p38 in T cells subsets from healthy individuals (HI) and end stage renal disease (ESRD) patients. Phosphorylation of p38 in (**a**) CD4^+^, (**b**) CD4^+^ naive, (**c**) CD4^+^ central memory (CM), (**d**) CD4^+^ effector memory (EM), as well as (**e**) CD8^+^, (**f**) CD8^+^ naive, (**g**) CD8^+^ CM, (**h**) CD8^+^EM, (**i**) CD8^+^ highly differentiated effector T cells (EMRA). *Dots* and *squares* represent young (*n* = 13) and elderly HI (*n* = 11), and *upward*- and *downward-facing triangles* correspond to young (*n* = 12) and elderly (*n* = 12) patients, respectively. *P* value: *< 0.05; Data are given as median with interquartile range

However, no significant differences in expression levels of pERK in total CD4^+^ T cells or the subsets were found comparing young and elderly ESRD patients (Fig. 2a–c &d). For example, the median (interquartile range)) MFI value of CD4^+^ pERK in young patients was 613 (490–664) and 541 (413–801) in elderly patients (*p* = 0.51). The median MFI value for CD4^+^ pERK in young patients was in between the MFI values of young HI 658 (485–1212)) and elderly HI (535 (305–620)) but did not significantly differ from either group (respectively *p* = 0.13 and *p* = 0.06). This age-associated decline in pERK was not found for CD8^+^ T cell subsets (Fig. 2e–h & i). In addition, an age-related decline in TCR-mediated phosphorylation of p38, was absent in CD4^+^ T cells (Fig. 3a–c & d) as well as CD8^+^ T cells (Fig. 3e–h & i) of both HI and ESRD patients. In conclusion, ESRD patients have a defective ERK, but not p38, phosphorylation in CD4^+^ T cells, that is independent of age and at a level similar to aged HI.

### Phosphorylation of ERK is associated with T-cell differentiation status

**Fig. 4:**
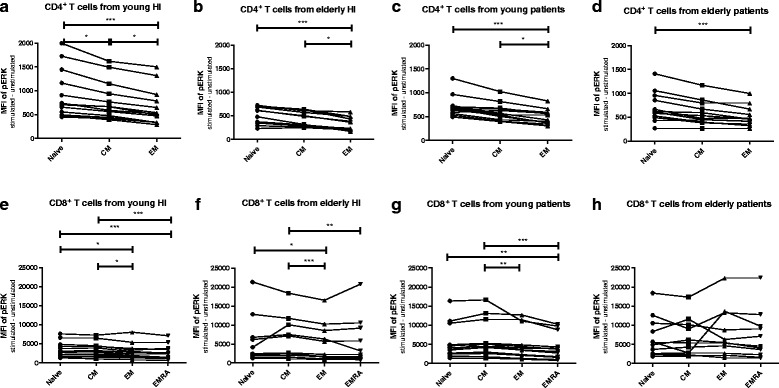
Phosphorylation of ERK according to T cell differentiation status in healthy individuals (HI) and end stage renal disease (ESRD) patients. Phosphorylation of ERK in CD4^+^ T cells from (**a**) young HI (*n* = 13), (**b**) elderly HI (*n* = 11), (**c**) young patients (*n* = 12), and (**d**) elderly patients (*n* = 12), as well as CD8^+^ T cells from (**e**) young HI, (**f**) elderly HI, (**g**) young patients, and (**h**) elderly patients. *Dots*, *squares*, *upward*- and *downward-facing triangles* represent naive, central memory (CM), effector memory (EM) and highly differentiated effector T cells (EMRA) T cells, respectively. *P* value: *< 0.05; **< 0.01; ***< 0.001; Data are given as median with interquartile range

**Fig. 5:**
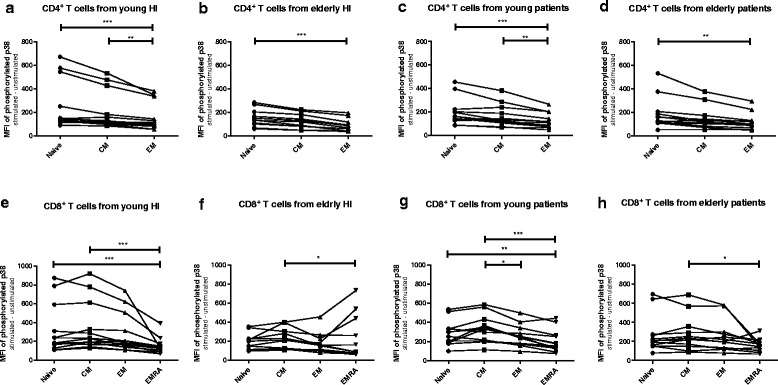
Phosphorylation of p38 according to T cell differentiation status in healthy individuals (HI) and end stage renal disease (ESRD) patients. Phosphorylation of ERK in CD4^+^ T cells from (**a**) young HI (*n* = 13), (**b**) elderly HI (*n* = 11), (**c**) young patients (*n* = 12), and (**d**) elderly patients (*n* = 12), as well as CD8^+^ T cells from (**e**) young HI, (**f**) elderly HI, (**g**) young patients, and (**h**) elderly patients. *Dots*, *squares*, *upward*- and *downward-facing triangles* represent naive, central memory (CM), effector memory (EM) and highly differentiated effector T cells (EMRA) T cells, respectively. *P* value: *< 0.05; **< 0.01; ***< 0.001; Data are given as median with interquartile range

Next, we compared phosphorylation of ERK and p38 within different T cell subsets to assess whether differentiation-associated effects exist in the study groups. In all groups, a gradual decrease in TCR-induced phosphorylation capacity was seen with increasing CD4^+^ T cell differentiation. Phosphorylation of ERK was highest in naive CD4^+^ T cells of young HI, followed by that in the CM and EM subsets of the memory compartment (Fig. 4a). Median MFI dropped from 722 to 666 and 517 in the naive, CM and EM T cell subset, respectively. Interestingly, in elderly HI as well as both groups of ESRD patients (Fig. 4b, c & d), pERK levels were still highest within naive CD4^+^ T cells compared to the more differentiated EM T cell subset, but the difference with that observed within CM T cells disappeared. ERK phosphorylation within CM is higher than that within the EM compartment in young and elderly HI (Fig. 4a & b), as well as in young patients (Fig. 4c), but not in elderly patients (Fig. 4d). Differences for the various CD8^+^ T-cell subsets with respect to TCR-mediated phosphorylation of ERK between naive and CM compartment, or between EM and EMRA were less outspoken and not significantly different in HI (Fig. 4e & f) and patients (Fig. 4g & h). Similar to ERK, phosphorylation of p38 showed a similar trend to decrease with increasing differentiation status but no significant decline in phosphorylation of p38 from naive to CM in CD4^+^ in HI and patients (Fig. 5a–c & d). In CD8^+^ T cells, p38 phosphorylation was decreased in highly differentiated EMRA compared to CM in HI and patients (Fig. 5e–g & h). In conclusion, a differentiation-associated decrease in anti-CD3/CD28-induced phosphorylation of ERK and p38 in T cells was present in HI and patients.

### Auto-phosphorylation (baseline) of ERK was associated with ageing in HI and increased according to differentiation status in CD4^+^ T cells

**Fig. 6:**
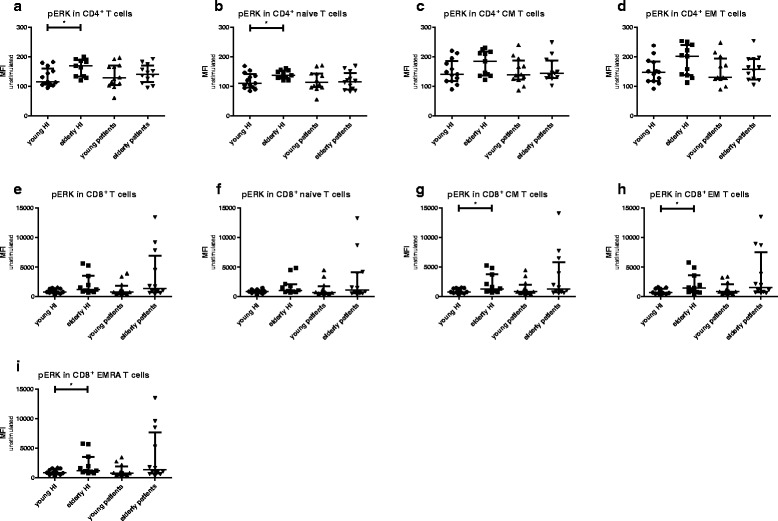
Auto-phosphorylation (baseline) of ERK in T cells subsets from healthy individuals (HI) and end stage renal disease (ESRD) patients. Phosphorylation of ERK in (**a**) CD4^+^, (**b**) CD4^+^ naive, (**c**) CD4^+^ central memory (CM), (**d**) CD4^+^ effector memory (EM), as well as (**e**) CD8^+^, (**f**) CD8^+^ naive, (**g**) CD8^+^ CM, (**h**) CD8^+^EM, (**i**) CD8^+^ highly differentiated effector T cells (EMRA). *Dots* and *squares* represent young (*n* = 13) and elderly HI (*n* = 11), and *upward*- and *downward-facing triangles* correspond to young (*n* = 12) and elderly patients (*n* = 12), respectively. *P* value: *< 0.05; Data are given as median with interquartile range

**Fig. 7:**
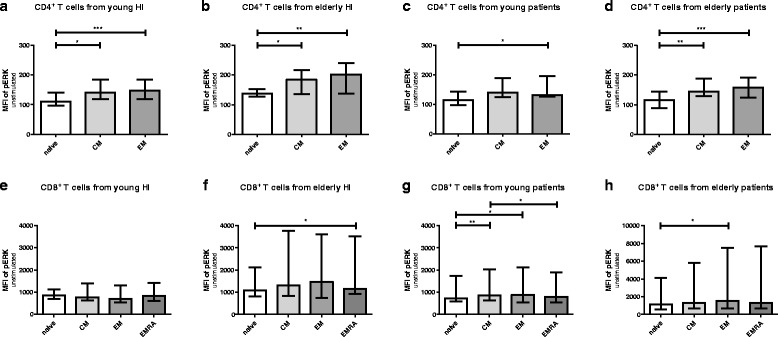
Auto**-**phosphorylation (baseline) of ERK according to T cell differentiation status in healthy individuals (HI) and end stage renal disease (ESRD) patients. Phosphorylation of ERK in CD4^+^ T cells from (**a**) young HI (*n* = 13), (**b**) elderly HI (*n* = 11), (**c**) young patients (*n* = 12), and (**d**) elderly patients (*n* = 12), as well as CD8^+^ T cells from (**e**) young HI, (**f**) elderly HI, (**g**) young patients, and (**h**) elderly patients. *Blank bars* and *bars* with *light grey* to *dark grey* represent naive, central memory (CM), effector memory (EM) and highly differentiated effector T cells (EMRA) T cells, respectively. *P* value: *< 0.05; **< 0.01; ***< 0.001; Data are given as median with interquartile range

Baseline phosphorylation of ERK (i.e. auto-phosphorylation) was significantly lower for total and naive CD4^+^T cells in young HI compared with elderly HI, however, this trend was not observed in patients (Fig. 6a & b). Baseline levels of pERK were significantly lower in the CD8^+^ memory compartment (CM, EM and EMRA) when comparing young to elderly HI, but no age-related differences were observed for patients (Fig. 6g, h & i). Baseline phosphorylation of ERK was lower in the naive CD4^+^ T cells when compared to CM and/or EM in HI (Fig. 7 a & b) and patients (Fig. 7c & d). Naive CD8^+^ T cells also had lower baseline pERK when compared to EMRA in elderly HI (Fig. 7f), CM and EM in young patients (Fig. 7g), and EM in elderly patients (Fig. 7h). Naive CD4^+^ T cells had lower baseline phosphorylated p38 compared to CM or EM in elderly HI (Additional file 1: Fig. S1b), and lower p38 phosphorylation compared to EM in young patients (Additional file 1: Figure S1c). In CD8^+^ T cells, p38 auto-phosphorylation in naive compartment was significantly lower than that of EMRA in young and elderly HI (Additional file 1: Figure S1e & f), but this trend was not observed in the patient population (Additional file 1: Figure S1 g & h). To summarize, an age- as well as differentiation-related increase in baseline levels of pERK was observed for CD4^+^ T cells.

### Neither CMV-serostatus nor RRT significantly influenced ERK or p38 phosphorylation

CMV-seropositivity may promote immunological T-cell ageing and was therefore analyzed for its association with MAPK pathway activation. However, ERK or p38 phosphorylation within T cells showed no significant difference comparing CMV-seropositive individuals to their CMV- seronegative counterparts in healthy individuals and ESRD patients respectively (Additional file 1: Figure S2 & S3). Moreover, in ESRD patients, ERK or p38 phosphorylation was not significantly different between the patients receiving RRT (i.e. dialysis) and those without RRT (Additional file 1: Fig. S4 & S5). In conclusion, neither CMV-serostatus nor RRT significantly influenced TCR-stimulation induced phosphorylation of ERK or p38.

### Age-related decline in anti-CD3/CD28-induced CD69-expressing CD4^+^ T cells positively associated with phosphorylation of ERK

**Fig. 8:**
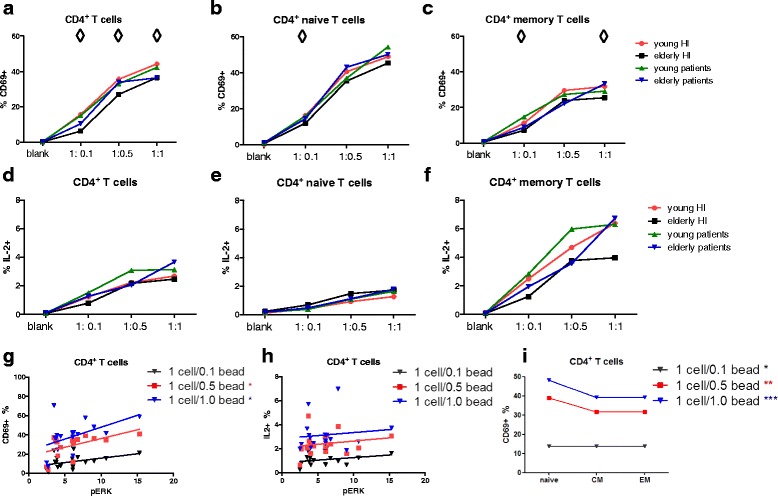
Percentages of CD69-expressing and IL-2 producing CD4^+^ T cell subsets in healthy individuals (HI) and end-stage renal disease (ESRD) patients. Percentages of CD69^+^ CD4^+^T cells were shown from healthy individuals (HI) (young *n* = 4, elderly *n* = 5) and end-stage renal disease (ESRD) patients (young *n* = 4, elderly *n* = 5) for (**a**) total, (**b**) naive, (**c**) memory CD4^+^ T cells, and frequencies of IL-2 expression were given for (**d**) total, (**e**) naive, (**f**) memory CD4^+^ T cells. *Red dots* and *orange squares* represent young and elderly HI, and *blue upward*- and *purple downward-facing triangles* correspond to young and elderly patients, respectively. The association between (**g**) percentages of CD69^+^ or (**h**) IL-2^+^ CD4^+^ T cells and ERK phosphorylation (depicted as fold increase by dividing the MFI of stimulated cells by that of unstimulated cells) is depicted. (**i**) The differentiation-associated relation between percentages of CD69^+^ and different CD4^+^ T cell subsets (naive, CM, EM) is shown. *Black* and *blue downward-facing triangles* represent 1 cell/0.1 bead and 1 cell/1 bead stimulation, and red squares correspond to 1cell/0.5 bead stimulation. ◊ represents a significant difference in the percentage of CD69^+^ T cells calculated for each specific CD4^+^ T cell subset when comparing young with elderly HI, or when comparing young with elderly patients. *represents a significant difference in the percentage of CD69^+^ T cells comparing CD4^+^ subsets or CD8^+^ subsets within each study group. *P* value: ◊ <0.05;*< 0.05; **< 0.01; ***< 0.001. Data are given as medians

A typical flow cytometric example for analysis of frequencies of CD69-expressing and IL-2 producing CD4^+^ T cells after CD3/CD28 stimulation was given in Additional file 1: Figure S6. Higher frequencies of CD69-expressing CD4^+^ T cells were observed in young HI compared to that in elderly HI when stimulated with different ratios of anti-CD3/CD28 beads to cells (Fig. 8a). This age-related decline in CD69-expressing cells was observed for naive CD4^+^ T cells in the healthy population at a ratio of 1:0.1 (Fig. 8b) and at 1:0.1 and 1:1 ratios for memory CD4^+^ T cells (Fig. 8c). Percentages of IL-2 producing CD4^+^ T cells did not reveal an age-associated decline in our study population (Fig. 8d, e & f). Percentages of CD69-expressing CD4^+^ T cells following stimulation with anti-CD3/CD28 beads (at ratios 1:0.5 and 1:1) were associated with ERK phosphorylation, presented as the fold increase in MFI dividing the MFI from stimulated samples by that of unstimulated samples (Fig. 8g). Percentages of IL-2 producing CD4^+^ T cells were not associated with pERK (Fig. 8h). In addition, frequencies of CD69-expressing CD4^+^ T cells were significantly higher in the naive subset compared to CM or EM (Fig. 8i). In short, an age-related decline in anti-CD3/CD28-induced percentages of CD69-expressing CD4^+^ T cells was observed and percentages of CD69-expressing CD4^+^ T cells were positively associated with phosphorylation of ERK.

### BCI promoted TCR-mediated phosphorylation of ERK

**Fig. 9:**
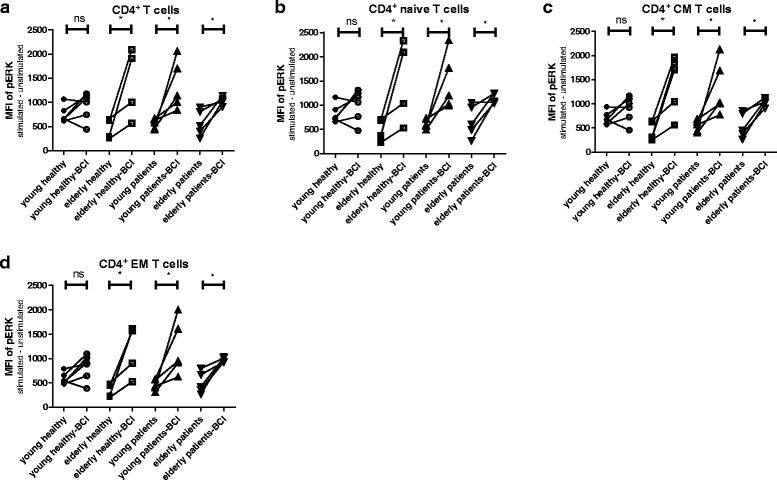
Phosphorylation of ERK in CD4^+^ T cell subsets without and with BCI treatment from healthy individuals (HI) and end stage renal disease (ESRD) patients. Phosphorylation of ERK for BCI-pretreated or not BCI-pretreated cells is given for different CD4^+^ T cell subsets: (**a**) total, (**b**) naive, (**c**) central memory (CM) and (**d**) effector memory (EM) of HI (young *n* = 5; elderly *n* = 5) and ESRD patients (young *n* = 5; elderly *n* = 5). *Dots* and *squares* represent young and elderly HI, upward- and downward-facing triangles correspond to young and elderly patients, respectively. *P* value: *< 0.05; Data are given as individual values

BCI is known to inhibit DUSP6 but also decrease levels of DUSP1 (product document, Merck – Millipore). BCI did not significantly enhance phosphorylation of ERK in young HI; in contrast, in elderly HI and both young and elderly ESRD patients, the pERK level was significantly upregulated in all CD4^+^ T cell subsets pretreated with BCI compared to that without (Fig. 9a–c & d). The average fold increase in pERK levels in naive CD4^+^ T cell subsets were 3.8, 2.5, and 2.1 in elderly HI, young and elderly ESRD patients, respectively. This was not observed for CD8^+^ T cells (Additional file 1: Figure S7). In short, BCI promoted TCR-mediated phosphorylation of ERK in CD4^+^ T cells of both elderly HI as well as young and old ESRD patients.

### DUSP6 and DUSP1 expression in CD4^+^ T cells

**Fig. 10:**
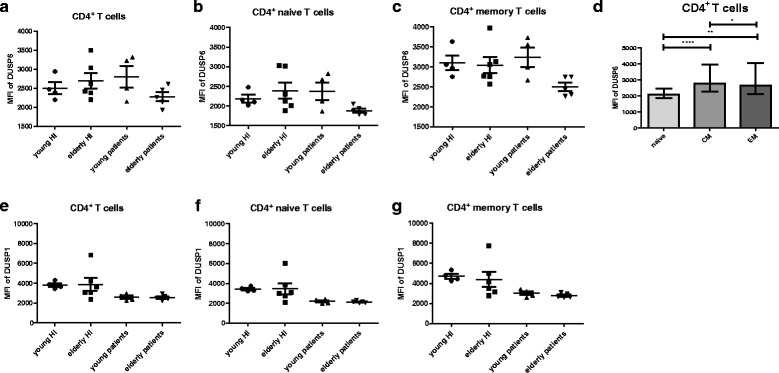
DUSP6 and DUSP1 expression in CD4^+^ T cell subsets from healthy individuals (HI) and end stage renal disease (ESRD) patients. DUSP6 expression in (**a**) total, (**b**) naive and (**c**) memory CD4^+^ T cells; (**d**) Differentiation-associated DUSP6 expression in CD4^+^ T cells; DUSP1 expression in (**e**) total, (**f**) naive and (**g**) memory CD4^+^ T cells; *Dots* and *squares* represent young (*n* = 4) and elderly HI (*n* = 6), and *upward*- and *downward-facing triangles* correspond to young (*n* = 4) and elderly (*n* = 5) patients, respectively. *P* value: *< 0.05; **< 0.01; ***< 0.001; Data are given as (individual values and) medians with interquartile ranges

In an attempt to unravel whether effects of BCI on pERK levels were mediated by interfering with DUSP6 and/or DUSP1, we measured DUSP6 and DUSP1 levels within CD4^+^ T cells in a small fraction of our study cohort. Due to limited availability of materials only 10 HI (4 young and 6 elderly) and 9 ESRD patients (4 young and 5 elderly) could be included. A typical flow cytometric example for analysis of DUSP6 and DUSP1 is given in Additional file 1: Figure S8. An age-associated trend of increased levels of DUSP6 was noted for total and naive CD4^+^ T cells in HI (Fig. 10a & b). The opposite was present comparing young to old ESRD patients (Fig. 10a, b & c)**.** Levels of DUSP6 in young ESRD patients were similar to old HI. Like observed for pERK, a significant differentiation-associated increase in DUSP6 levels was observed (Fig. 10 d). Interestingly, DUSP1 expression in CD4^+^ T cells was quite comparable between both young and elderly HI and patients (Fig. 10e, f, & g). To conclude, an age-related tendency of increased levels of DUSP6, but not DUSP1, was observed for HI. Moreover, DUSP6 expression was significantly associated with T-cell differentiation status in CD4^+^ T cells.

## Discussion

The main observation of this study was that TCR-mediated phosphorylation of ERK in CD4^+^ T cells of young patients was in between young and old HI. Phosphorylation of ERK decreased in highly differentiated T-cell subsets compared to naive T cells. This defective TCR-mediated phosphorylation was specific as it could be restored by addition of a DUSP6 inhibitor. TCR-induced p38 phosphorylation was comparable between ESRD patients and HI.

Beyond midlife, the immune system shows age-related features and its defensive capabilities becomes impaired [35]. The uremia-associated inflammatory environment present in ESRD patients accelerates this age-related immune senescence process. In addition to declined thymic output, accumulation of highly differentiated T cells, short telomere length [10, 33, 36] and narrowed TCR-Vβ repertoire diversity [32], this study indicates that young ESRD patients also have a defective CD4^+^ TCR activation judging from the reduced capacity to phosphorylate ERK upon TCR-triggering. ERK activity is critical for TCR threshold calibration, as it controls positive feedback loops in TCR-induced activation [17]. Reduced ERK activity impairs TCR signal strength and activation, and favors T cells with higher affinity to antigen to be activated, leading to a contracted immune response to a given antigen [16]. The ERK phosphorylation upregulation of early activation marker CD69 on T cells ensures a proper inducing activation of T cells in the lymph node [37], and also play an important role in T cell proliferation [38] and IL-2 production [39, 40]. In addition, ERK activation impacts cellular apoptosis as it inhibits Fas-mediated apoptosis in T cells [41]. Evaluating ERK phosphorylation is a valuable tool to study more upstream molecules in the defective T-cell mediated immune system from ESRD patients. T cells from rheumatoid arthritis (RA) patients exhibit several defects which can also be viewed as premature immunological ageing [42]. However, ERK phosphorylation in CD4^+^ T cells of RA patients selectively increased [43]. This increased ERK activation lowers the TCR threshold in T cells of RA patients to respond to self-antigens, which may partly explain the adaptive immune system of RA patients to exhibit abnormalities that go beyond the local inflammatory response in the synovium [44].

DUSP6 is a cytoplasmic phosphatase with substrate specificity for phosphorylated ERK. In elderly individuals, silencing of DUSP6 increased the expression of T cell activation markers, such as CD69 and CD25, IL-2 production as well as proliferative response [16]. Inhibition of DUSP6 could be a potential intervention to increase CD4^+^ TCR-sensitivity by enhancing ERK phosphorylation in ESRD patients. BCI (an inhibitor of DUSP6 and 1) enhanced TCR-induced pERK in CD4^+^ T cells from elderly HI, young and elderly ESRD patients, but not young HI, implying a role for DUSP6 and/or DUSP1 in regulation of ERK phosphorylation. Based on the age- as well as differentiation-related expression of DUSP6, but not DUSP1, in our HI, a potential role for DUSP6 may be present in defective TCR-induced ERK phosphorylation, especially in elderly HI and young patients. This needs to be confirmed in a larger cohort. Furthermore, use of siRNA specific for DUSP6 is required to draw a more definite conclusion with respect to the role of DUSP6 in defective TCR-induced phosphorylation of ERK in ESRD patients. The lack of age-related effects on pERK in CD8^+^ T cells of ESRD patients and HI, the latter confirming observations done by another study [16], as well as absence of effects of BCI on pERK levels in CD8^+^ T cells, indicates a different role for DUSP6 in CD8^+^ T cells compared to CD4^+^ T cells. We did not observe an association between DUSP6 expression and ERK phosphorylation in CD4^+^ T cells in ESRD patients. This could be due to the small cohort size or imply other DUSPs (e.g. 2, 4 or 5) [45–49] or upstream signaling molecules to contribute to this defective TCR-induced ERK phosphorylation in ESRD patients.

ERK over-phosphorylation might be as bad as defective ERK phosphorylation. ERK over-activation from kidney cells occurs in the physiologic setting in some chronic kidney diseases, such as compensatory kidney hypertrophy and in pathologic conditions for example glomerular disease [50]. Increased ERK phosphorylation in T cells predisposes for autoimmunity for example rheumatoid arthritis [43]. Over-expression of DUSP6 is also reported to impair T-cell function in chronic viral infections such as hepatitis C virus infection [51]. Therefore, more research is warranted evaluating inhibition of DUSP in the setting of defective T-cell mediated immunity in ESRD patients.

We analyzed the effect of latency for CMV as it represents chronic antigenic stimulation of T cells, but ERK- or p38-activation of CD4^+^ or CD8^+^ T cells was not different between the CMV-IgG seropositive population and CMV-IgG seronegative population following CD3/CD28 stimulation. Highly differentiated memory CD4^+^ and CD8^+^ T cells may accumulate in CMV seropositive individuals and are functional CMV-specific T cells [29, 52, 53]. The results of our study show that non-specific TCR stimulation does not identify a defect p38 and ERK signaling associated with CMV seropositivity. In accordance with the results of a previous study, uremia is the major determinant affecting MAPK pathway parameters in ESRD patients and not RRT [9, 11, 33].

In the present study, we induced phosphorylation of p38 in T cells via triggering CD3 [54] and CD28 [26]. Lack of CD28 may only partly explain the decreased p38 activation in more differentiated CD4^+^ T cells. In addition to that, senescent human CD27^−^CD28^−^ CD4^+^ T cells lack several essential upstream components including ZAP70 and the loss of TCR signaling machinery in those cells was associated with a defective calcium influx [31], which may indicate the decreased response of TCR-mediated activation in the more differentiated T cells. Interestingly, in contrast to the p38 activation following CD3/CD28 stimulation, baseline levels (spontaneous phosphorylation) of p38 increased during T cells differentiation [55]. This might be caused by DNA damage in these more differentiated T cells and mediated by TAB1 (MKK-independent molecule), a key molecule involved in this auto-phosphorylation [31].

## Conclusion

We have described for the first time a uremia-mediated defect in TCR-induced phosphorylation of ERK which may contribute to the impaired T-cell mediated immune response in ESRD patients. Inhibition of DUSP6 specific for pERK can restore defective p-ERK-mediated activation of CD4^+^ T cells in ESRD patients.

## Additional files

Additional file 1: Figure S1. Auto-phosphorylation (baseline) of p38 according to T cell differentiation status in healthy individuals (HI) and end stage renal disease (ESRD) patients. **Figure S2**. CMV-effects on phosphorylation of ERK in T cells subsets of healthy individuals (HI) and end stage renal disease (ESRD) patients. **Figure S3**. CMV-effects on phosphorylation of p38 in T cells subsets of healthy individuals (HI) and end stage renal disease (ESRD) patients. **Figure S4**. Effects of renal replacement therapy (RRT) on phosphorylation of ERK in T cells subsets of end stage renal disease (ESRD) patients with and without dialysis. **Figure S5**. Effects of renal replacement therapy (RRT) on phosphorylation of p38 in T cells subsets of end stage renal disease (ESRD) patients with and without dialysis. **Figure S6**. Typical example of the gating strategy for analysis of percentages of CD69^+^ CD4^+^ T cell subsets. **Figure S7**. Phosphorylation of ERK in CD8+ T cell subsets without and with BCI treatment from healthy individuals (HI) and end stage renal disease (ESRD) patients. **Figure S8**. Typical example of the gating strategy for analysis of DUSP6 and DUSP1 expression in CD4^+^ T cell subsets. (PDF 643 kb)

## Abbreviations

AF: Alexa Fluor
AMPK: 5′ adenosine monophosphate-activated protein kinase
APC: Allophycocyanin
BCI: (*E*)-2-benzylidene-3-(cyclohexylamino)-2, 3-dihydro-1 *H*-inden-1-one
BV: Brilliant Violet
CM: Central memory
CMV: Cytomegalovirus
DUSP: Dual specific phosphatase
EM: Effector memory
EMRA: Highly differentiated effector T cells
ERK: Extracellular signal-regulated kinase
ESRD: End-stage renal disease
FITC: Fluorescein isothiocyanate
HCM: Human culture medium
HI: Healthy individuals
IL-2: Interleukin-2
MAPKs: mitogen-activated protein kinases
MFI: Median fluorescence intensity
MKKs: MAPK kinases
PBMCs: Peripheral blood mononuclear cells
PE: Phycoerythrin
PerCP: Peridinin chlorophyll
pERK: Phosphorylation of ERK
RA: Rheumatoid arthritis
RRT: Renal replacement therapy
TAB1: TAK1-binding protein 1
TAK1: Transforming growth factor-β-activated protein kinase 1
TCR: T cell receptor
ZAP70: CD3 zeta-chain associated protein kinase of 70 kDa
