# Supplementary material for: pERK-dependent defective TCR-mediated activation of CD4+ T cells in end-stage renal disease patients
Source: Immun Ageing. 2017 Jun 19;14:14. doi: 10.1186/s12979-017-0096-1 (PMC5477144; doi:10.1186/s12979-017-0096-1)
Supplement: Supplementary file 1 — Auto-phosphorylation (baseline) of p38 according to T cell differentiation status in healthy individuals (HI) and end stage renal disease (ESRD) patients. Figure S2. CMV-effects on phosphorylation of ERK in T cells subsets of healthy individuals (HI) and end stage renal disease (ESRD) patients. Figure S3. CMV-effects on phosphorylation of p38 in T cells subsets of healthy individuals (HI) and end stage renal disease (ESRD) patients. Figure S4. Effects of renal replacement therapy (RRT) on phosphorylation of ERK in T cells subsets of end stage renal disease (ESRD) patients with and without dialysis. Figure S5. Effects of renal replacement therapy (RRT) on phosphorylation of p38 in T cells subsets of end stage renal disease (ESRD) patients with and without dialysis. Figure S6. Typical example of the gating strategy for analysis of percentages of CD69+ CD4+ T cell subsets. Figure S7. Phosphorylation of ERK in CD8+ T cell subsets without and with BCI treatment from healthy individuals (HI) and end stage renal disease (ESRD) patients. Figure S8. Typical example of the gating strategy for analysis of DUSP6 and DUSP1 expression in CD4+ T cell subsets. (PDF 643 kb) [file 12979_2017_96_MOESM1_ESM.pdf]

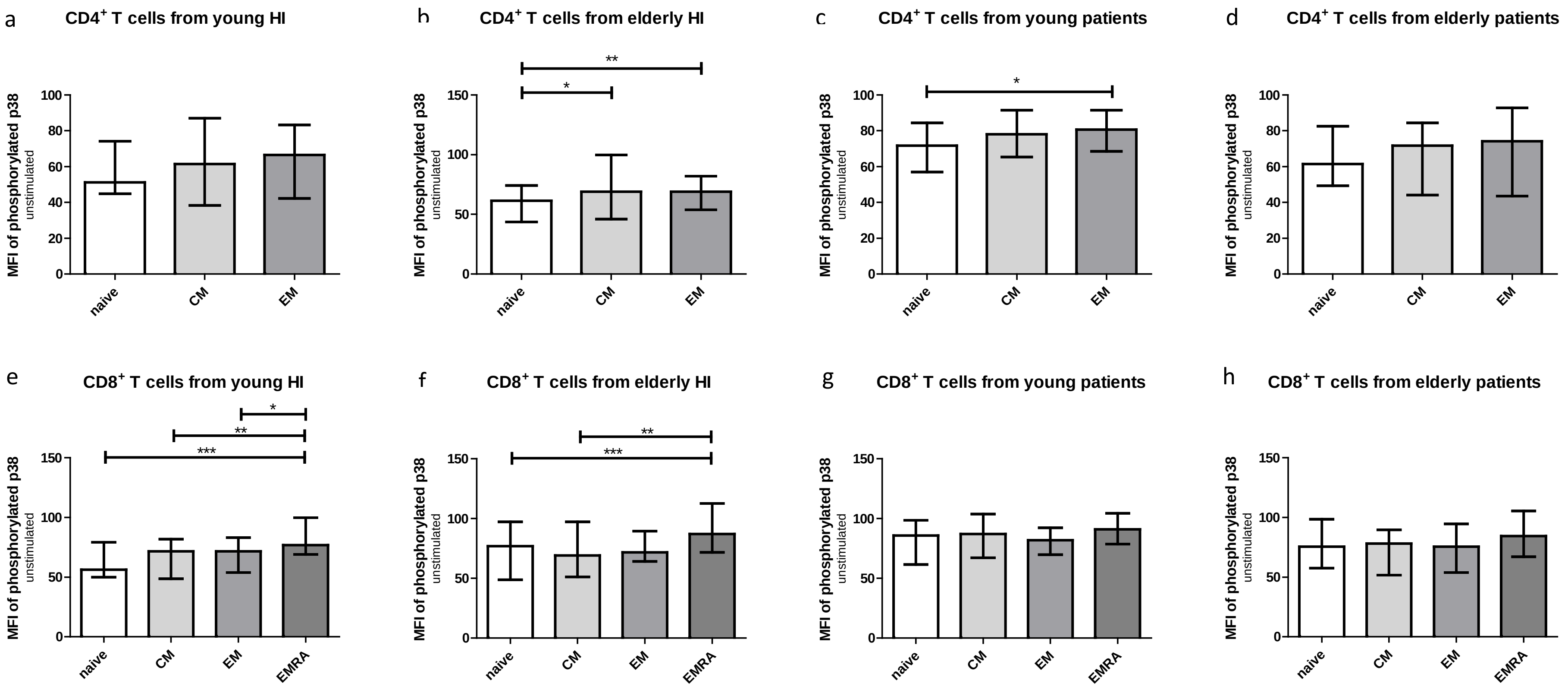

Figure S1. Auto-phosphorylation (baseline) of p38 according to T cell differentiation status in healthy individuals (HI) and end stage renal disease (ESRD) patients. Phosphorylation of ERK in CD4$^+$ T cells from (a) young HI (n=13), (b) elderly HI (n=11), (c) young patients (n=12), and (d) elderly patients (n=12), as well as CD8$^+$ T cells from (e) young HI, (f) elderly HI, (g) young patients, and (h) elderly patients. Blank bars and bars with light grey to dark grey represent naive, central memory (CM), effector memory (EM) and highly differentiated effector T cells (EMRA) T cells, respectively. P value: *<0.05; ** < 0.01; *** < 0.001; Data are given as median with interquartile range.

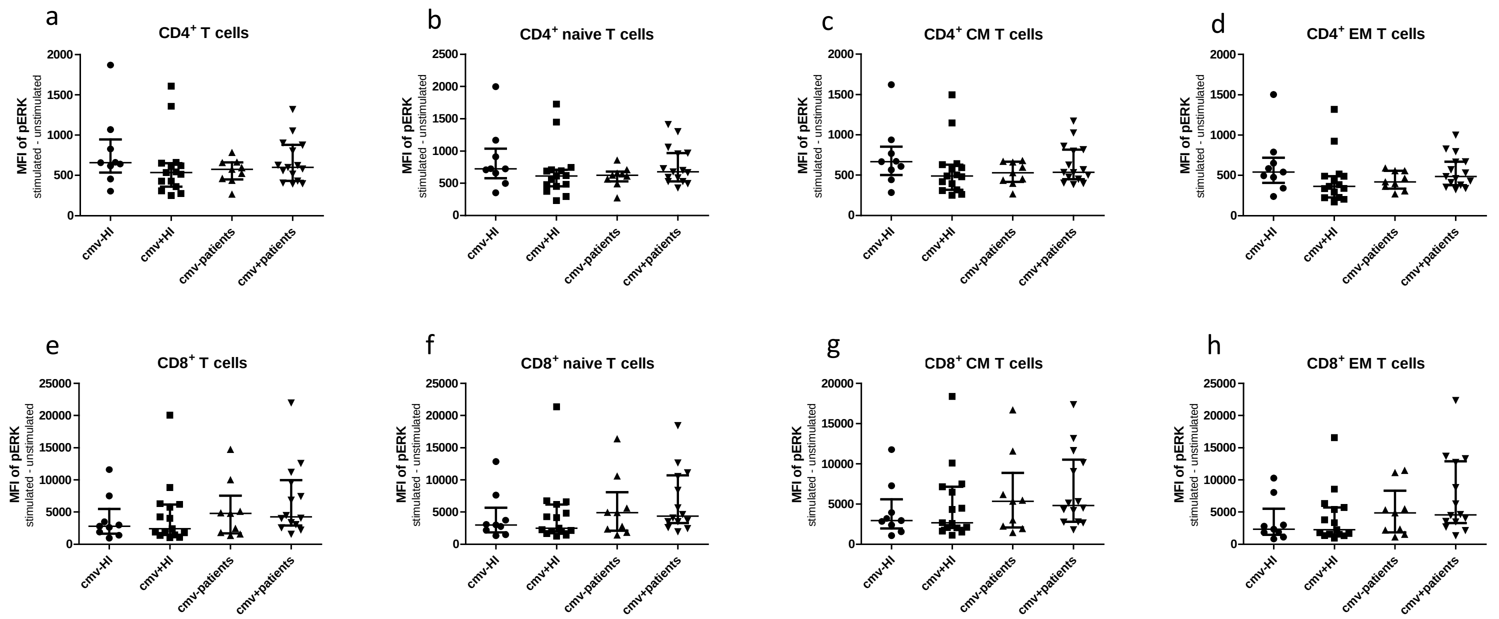

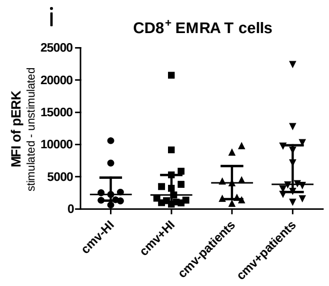

Figure S2. CMV-effects on phosphorylation of ERK in T cells subsets of healthy individuals (HI) and end stage renal disease (ESRD) patients. Phosphorylation of ERK (a) $CD4^+$, (b) $CD4^+$ naive, (c) $CD4^+$ central memory (CM), (d) $CD4^+$ effector memory (EM), as well as (e) $CD8^+$, (f) $CD8^+$ naive, (g) $CD8^+$ CM, (h) $CD8^+$EM, (i) $CD8^+$ highly differentiated effector T cells (EMRA). Dots and squares represent CMV seropositive (n=15) and negative (n=9) HI, upward- and downward-facing triangles correspond to CMV seropositive (n=15) and negative (n=9) patients, respectively. P value: * < 0.05; Data are given as individual values and medians with interquartile ranges .

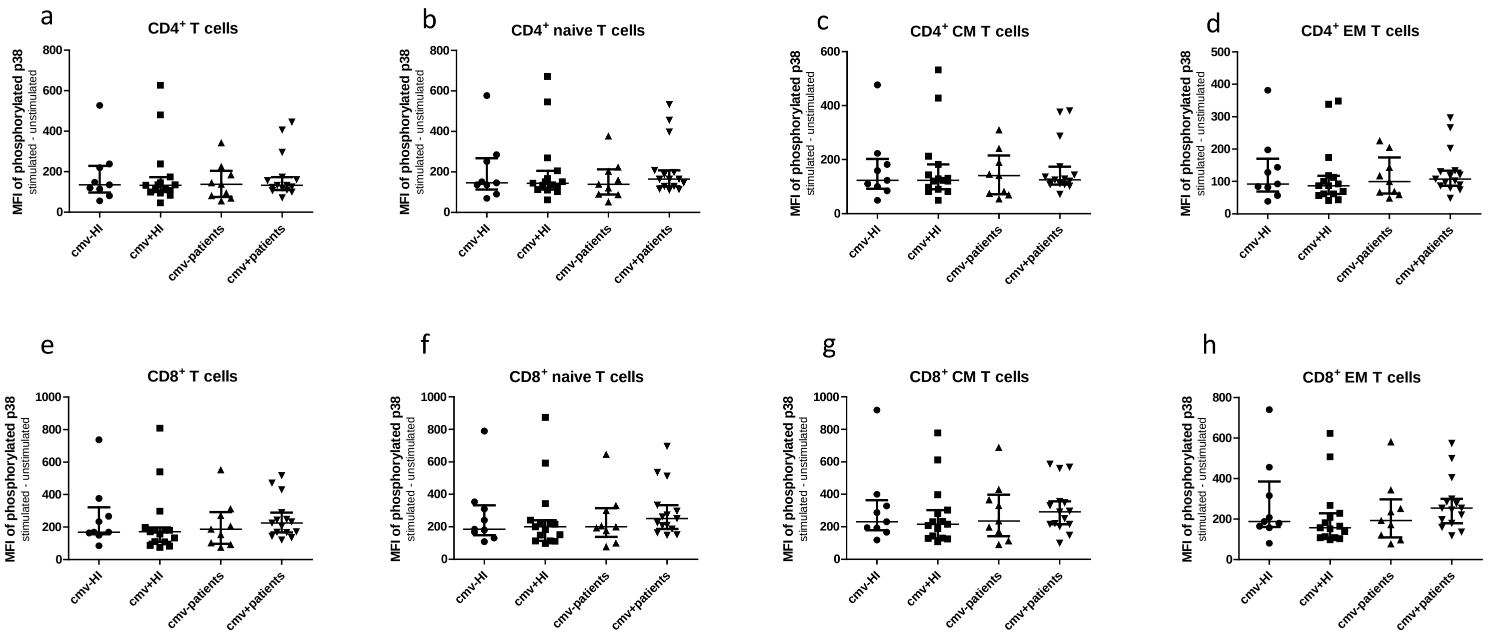

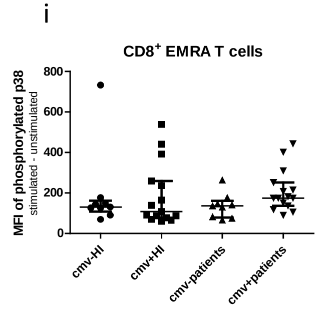

Figure S3. CMV-effects on phosphorylation of p38 in T cells subsets of healthy individuals (HI) and end stage renal disease (ESRD) patients. Phosphorylation of p38 (a) total, (b) naive, (c) central memory (CM), (d) effector memory (EM) $CD4^+$ T cells, as well as (e) total, (f) naive, (g) CM, (h) EM and (i) highly differentiated effector memory $CD45RA^+$ (EMRA) $CD8^+$T cells. Dots and squares represent CMV seropositive (n=15) and negative (n=9) HI, and upward- and downward-facing triangles correspond to CMV seropositive (n=15) and negative (n=9) patients, respectively. P value: * < 0.05; Data are given as individual values and medians with interquartile ranges.

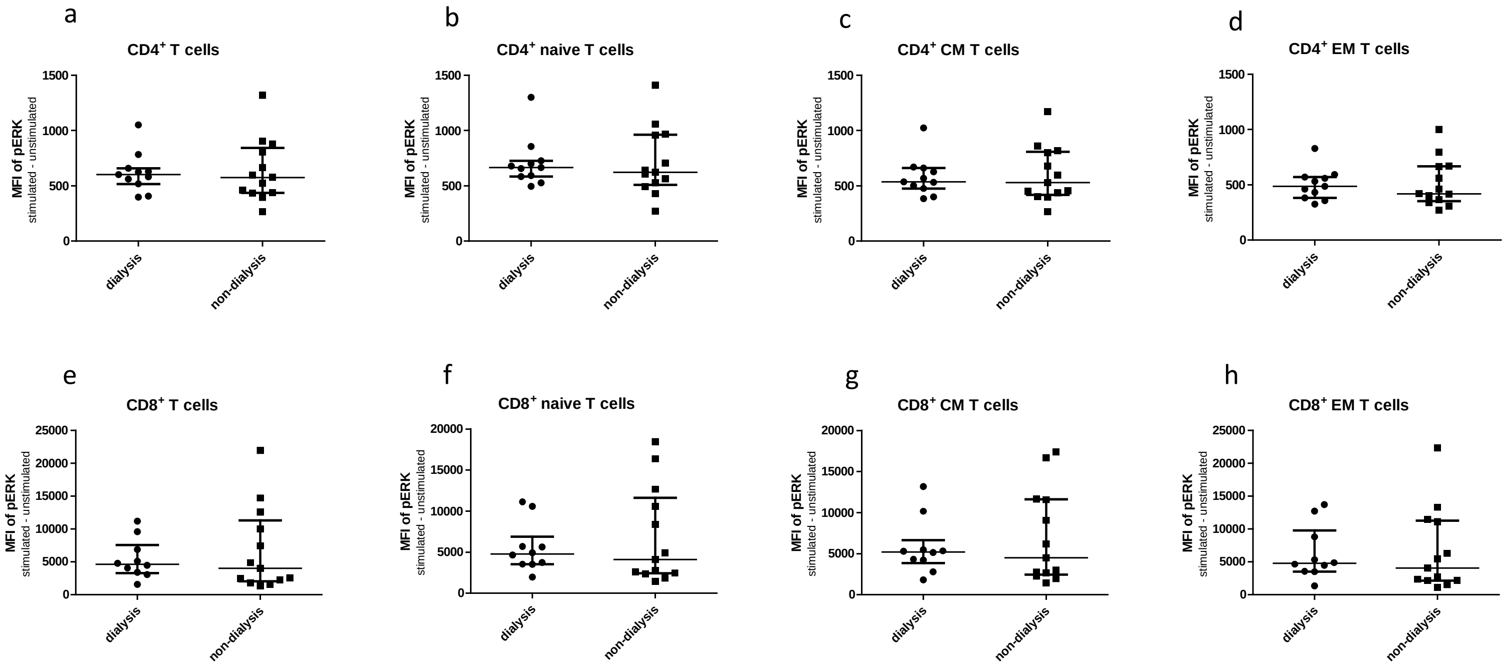

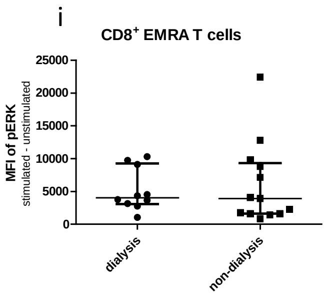

Figure S4. Effects of renal replacement therapy (RRT) on phosphorylation of ERK in T cells subsets of end stage renal disease (ESRD) patients with and without dialysis. Phosphorylation of ERK in (a) total, (b) naive, (c) central memory (CM), (d) effector memory (EM) $CD4^+$ T cells, as well as (e) total, (f) naive, (g) CM, (h) EM and (i) highly differentiated effector memory $CD45RA^+$(EMRA) $CD8^+$ T cells. Dots and squares represent patients with (n=11) and without dialysis (n=13), respectively. P value: * < 0.05; Data are given as medians with interquartile ranges.

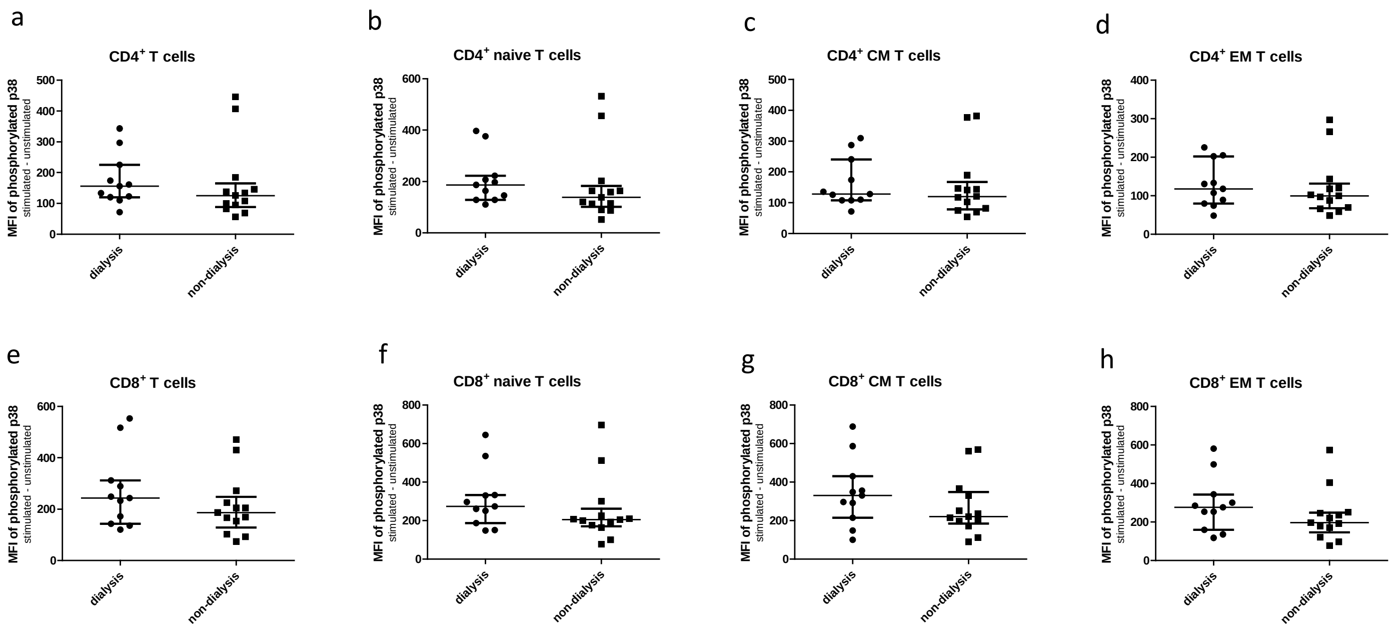

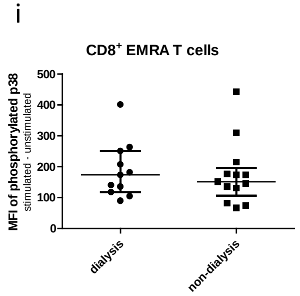

Figure S5. Effects of renal replacement therapy (RRT) on phosphorylation of p38 in T cells subsets of end stage renal disease (ESRD) patients with and without dialysis. Phosphorylation of p38 (a) total, (b) naive, (c) central memory (CM), (d) effector memory (EM) $CD4^+$ T cells as well as (e) total, (f) naive, (g) CM, (h) EM and (i) highly differentiated effector memory $CD45RA^+$ (EMRA) $CD8^+$ T cells. Dots and squares represent patients with (n=11) and without dialysis (n=13), respectively. P value: * < 0.05; Data are given as medians with interquartile ranges.

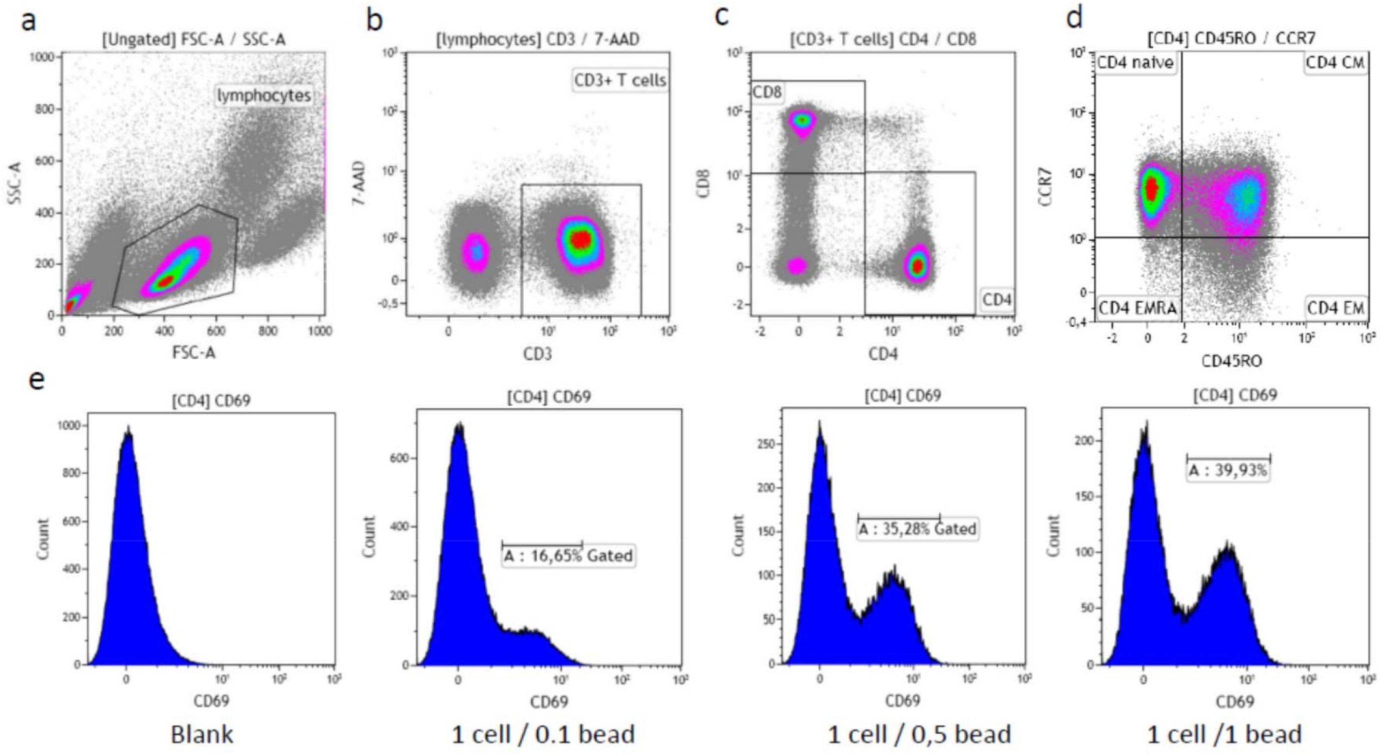

Figure S6. Typical example of the gating strategy for analysis of percentages of CD69$^+$ CD4$^+$ T cell subsets. Briefly, (a) lymphocytes were identified based on the forward/sideward characteristics followed by (b) the selection of CD3$^+$ living T cells. (c) These T cells were then dissected into CD4$^+$ and CD8$^+$ T cells. (d) CCR7 and CD45RO were used to identify naive and different memory subsets within CD4$^+$ T cells. Furthermore, (e) percentages of CD69$^+$ were measured for CD4$^+$ T cells stimulated or not with different ratios of anti-anti-CD3/CD28 beads (blank control, 1 cell /0.1 bead, 1 cell /0.5 bead, 1 cell/1 bead). A similar gating strategy was employed for analysis of CD69$^+$ and IL2$^+$ the different CD4$^+$ T cells subsets.

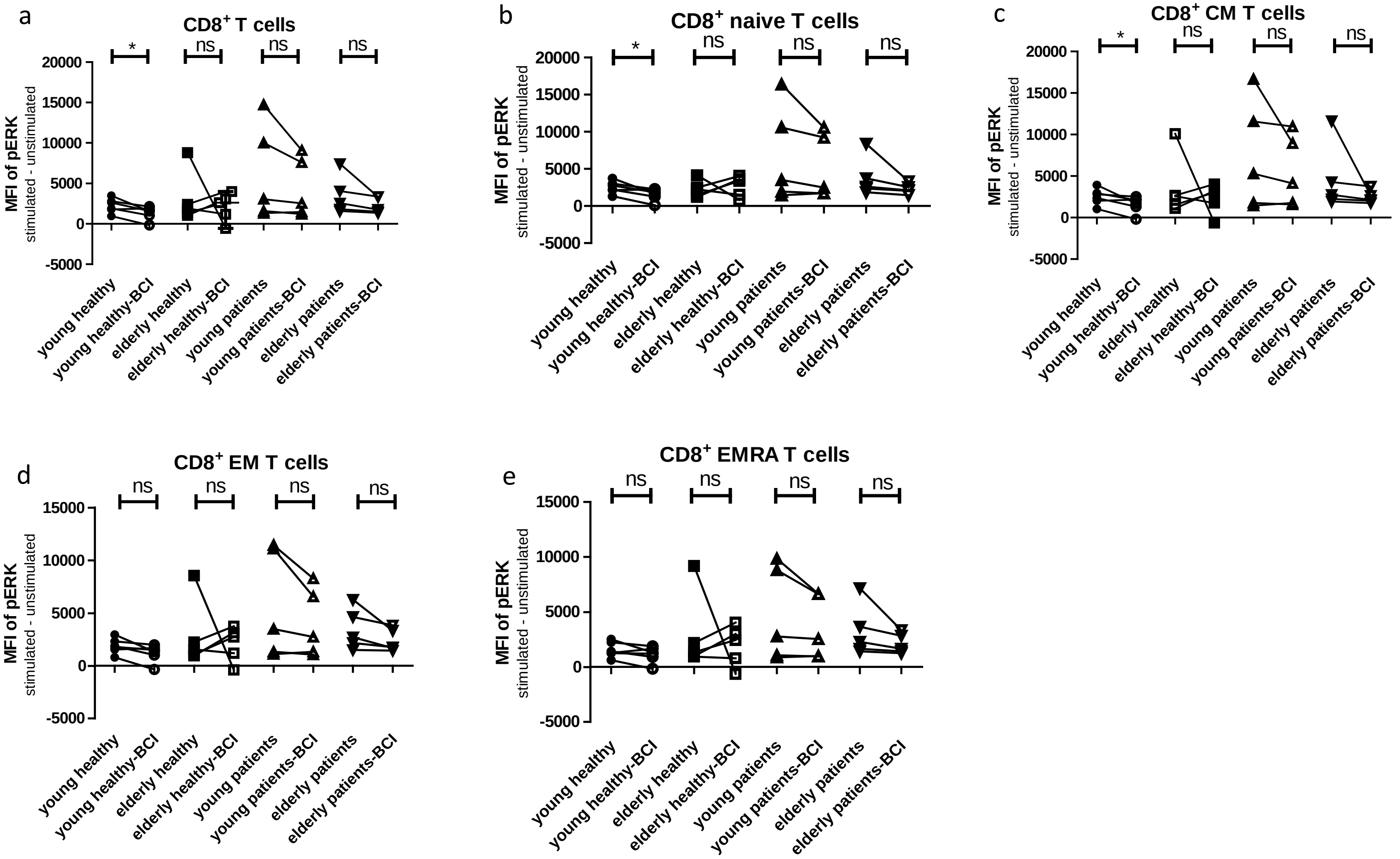

Figure S7. Phosphorylation of ERK in CD8$^+$ T cell subsets without and with BCI treatment from healthy individuals (HI) and end stage renal disease (ESRD) patients. Phosphorylation of ERK for BCI-pretreated or not BCI-pretreated cells is given for different CD4$^+$ T cell subsets: (a) total, (b) naive, (c) central memory (CM) and (d) effector memory (EM) of HI (young n=5; elderly n=5) and ESRD patients (young n=5; elderly n=5). Dots and squares represent young and elderly HI, upward- and downward-facing triangles correspond to young and elderly patients, respectively. P value: *<0.05; Data are given as individual values.

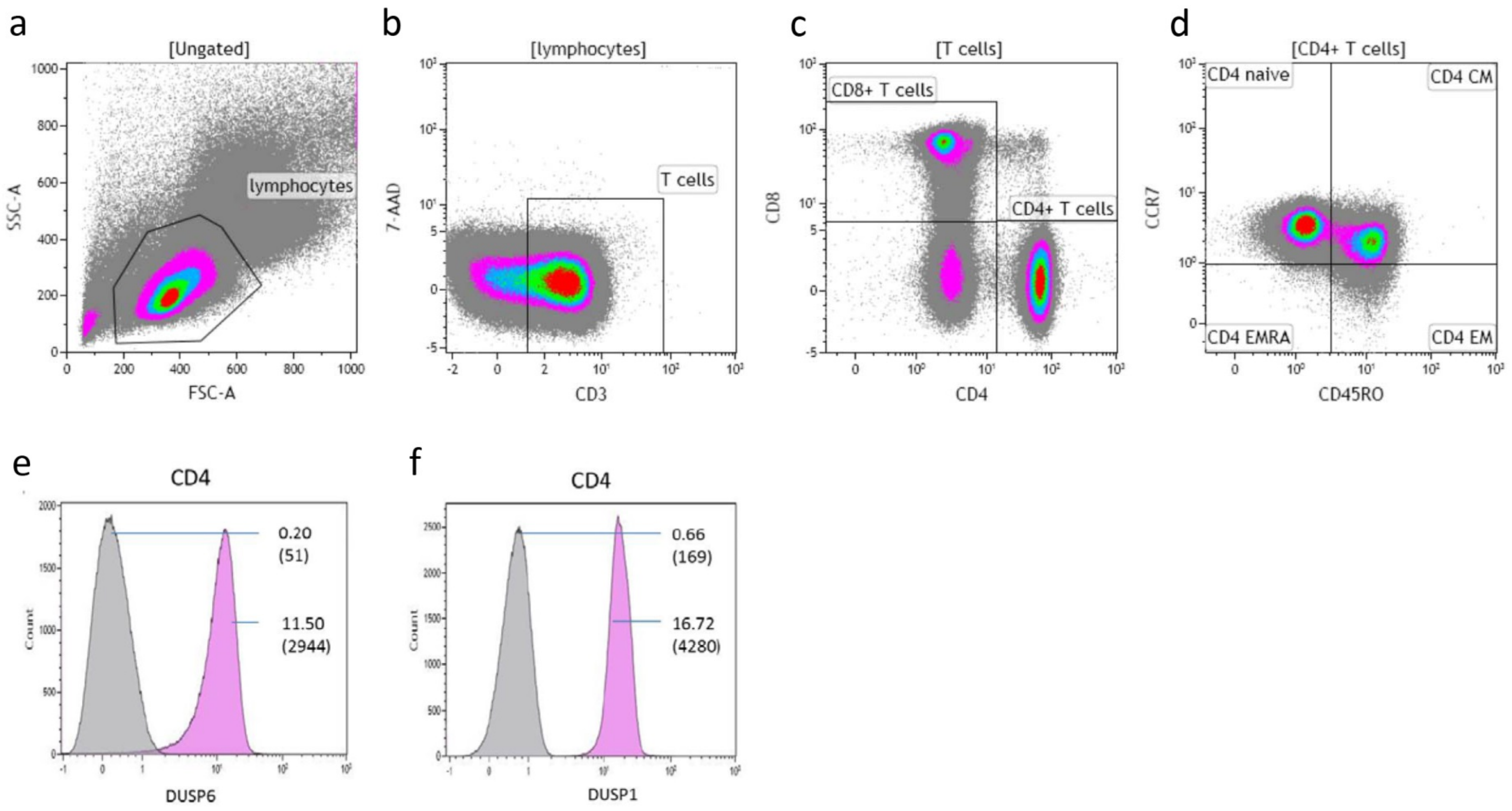

Figure S8. Typical example of the gating strategy for analysis of DUSP6 and DUSP1 expression in CD4$^+$ T cell subsets. Briefly, (a) lymphocytes were identified based on the forward/sideward characteristics followed by (b) the selection of CD3$^+$ living T cells. (c)These T cells were then dissected into CD4$^+$ and CD8$^+$ T cells. (d) CCR7 and CD45RO were used to identify naive and different memory subsets within CD4$^+$ T cells. Furthermore, (e) DUSP6 or (f) DUSP1 expression was measured in CD4$^+$ T cells and median fluorescence intensities (MFI) were evaluated (values multiplied by 256 in brackets). Grey peaks represent staining without the specific antibodies (fluorescence minus one, FMO), and pink peaks correspond to staining with antibodies directed to DUSP6 or DUSP1. A similar gating strategy was employed for analysis of DUSP6 and DUSP1 expression in all CD4$^+$ T cells subsets.
